# Polyphosphate application influences morpho-physiological root traits involved in P acquisition and durum wheat growth performance

**DOI:** 10.1186/s12870-022-03683-w

**Published:** 2022-06-27

**Authors:** Said Khourchi, Abdallah Oukarroum, Asma Tika, Pierre Delaplace, Adnane Bargaz

**Affiliations:** 1 Laboratory of Plant-Microbes Interactions, Agrobiosciences, Mohammed VI Polytechnic University, Ben Guerir, 43150 Rabat, Morocco; 2 Terra Department, Plant Sciences Group, Gembloux Agro-Bio Tech, Université de Liège, B-5030 Gembloux, Belgium

**Keywords:** Polyphosphate, P acquisition, Nutrition, Root traits, Rhizosphere, Photosynthesis

## Abstract

**Background:**

Among phosphate (P) fertilizers, polyphosphates (PolyPs) have shown promising results in terms of crop yield and plant P nutrition. However, compared to conventional P inputs, very little is known on the impact of PolyPs fertilizers on below- and above-ground plant functional traits involved in P acquisition. This study aims to evaluate agro-physiological responses of durum wheat variety ´Karim´ under different PolyPs applications. Three PolyPs fertilizers (PolyA, PolyB, and PolyC) *versus* one orthophosphate (OrthoP) were applied at three doses; 30 (D30), 60 (D60), and 90 (D90) kg P/ha under controlled conditions.

The PolyPs (especially PolyB and PolyC) application at D60 significantly increased morphophysiological root traits (e.g., RL: 42 and 130%; RSA:40 and 60%), shoot inorganic P (Pi) content (159 and 88%), and root P acquisition efficiency (471 and 296%) under PolyB and PolyC, respectively compared to unfertilized plants. Above-ground physiological parameters, mainly nutrient acquisition, chlorophyll content and chlorophyll fluorescence parameters were also improved under PolyB and PolyA application at D60. A significant and positive correlation between shoot Pi content and rhizosphere soil acid phosphatase activity was observed, which reveal the key role of these enzymes in PolyPs (A and B) use efficiency. Furthermore, increased P uptake/RL ratio along with shoot Pi indicates more efficient P allocation to shoots with less investment in root biomass production under PolyPs (especially A and B).

**Conclusions:**

Under our experimental conditions, these findings report positive impacts of PolyPs on wheat growth performance, particularly on photosynthesis and nutrient acquisition at D60, along with modulation of root morpho-physiological traits likely responsible of P acquisition efficiency.

**Supplementary Information:**

The online version contains supplementary material available at 10.1186/s12870-022-03683-w.

## Background

Generally, the low phyto-availability of nitrogen (N), phosphorus (P), or potassium (K) in many agricultural soils restricts crop production [1]. These nutrients are supplied to agricultural soils, among other forms, as mineral fertilizers. For more than a century, P has typically been supplied to agricultural soils as orthophosphate (OrthoP)-based fertilizers such as monoammonium P, triple superphosphate, and single superphosphate [2, 3].

However, the use efficiency of these fertilizers is still below expectations with less than 30% of applied P fertilizers being taken up by plants [4–6]. This is generally due to low P mobility and its strong adsorption and precipitation in the soil matrix, specifically in soil with a high content of metal ions such as Fe^3+^ and Al^3+^ [7–9].

To overcome this low P availability constraint and improve crop productivity, a focus on the rational application of more efficient P sources is urgently needed. Among this large range of P fertilizer types, polyphosphates (PolyPs) based fertilizers were used in agriculture and are known for their progressive hydrolysis in soils [10–12]. These characteristics make PolyPs fertilizers a sustainable P source that presumably would continuously release available P into the soil solution over time to fulfill plant requirements and reduce P loss in agricultural soils. In addition, PolyPs have been reported to chelate some essential micronutrients [e.g., iron (Fe), zinc (Zn), and manganese (Mn)], which is a characteristic that OrthoP does not have [13–17].

Only few studies have reported that PolyPs efficiency can be attributed to their higher and longer-term P availability during the whole crop growth period [9, 18–21]. Recently, Gao et al. [15] reported that ammonium PolyP significantly enhanced maize biomass (especially root biomass), P uptake, and P fertilizer use efficiency compared to plants fertilized with ammonium P. It is also reported a positive correlation between Olsen-P, maize dry weight, and P-uptake [15]. Furthermore, ammonium PolyP application not only had significantly increased P uptake, but also micronutrient availability (e.g., Fe, Mn, and Zn), which accounted for additional agronomic benefits of PolyPs [14, 15, 22, 23].

In terms of agronomic efficiency, many studies conducted under both greenhouse and field conditions, have shown that applications of PolyPs (different polymers of sodium PolyP, ammonium PolyP) to different staple crops (e.g., wheat, maize, chickpea, and soybean) increased grain yield, dry biomass, soil P availability, and P uptake [21, 24–27]. However, the beneficial effects that PolyPs may have on above-ground physiology (e.g., photosynthesis) and rhizosphere functioning – including root system development – are still poorly documented. For instance, specific root length, root hair length, root branching, and exudation of P-hydrolyzing enzymes into the rhizosphere are among the functional root features involved in P availability in the root vicinity and its uptake [20, 24, 28–33]. These findings provide strong evidence that these morpho-physiological root traits can be tightly linked to root growth and contribute to a gradual increase of P availability from PolyP. In this context, Dick and Tabatabai (1986) provided preliminary evidence that PolyPs may impact root functioning and growth, demonstrating that significant amounts of pyrophosphatases were produced by corn roots soaked in pyrophosphate solution. Based on the limited available findings in this research area, it can be assumed that below-ground traits (such as rhizosphere acidification and P-hydrolyzing enzyme exudation) could be new research directions to be exploited by scientists as a sustainable approach to enhance plant P use efficiency after PolyPs application. Given the lack of information on plant responses to PolyPs application, this study hypothesizes that PolyPs application at increasing P doses might yield differential responses in wheat growth and more particularly root development, which is essential for plant nutrient uptake, notably P. Specifically, the present study aimed to i) evaluate the effect of contrasted PolyPs application on wheat plants growth performance, ii) shed light, for the first time, on wheat belowground responses focusing on morphophysiological root traits presumably linked to P acquisition, and iii) decipher specific above-below-ground key connections supporting the hypothesis that PolyPs application can modulate wheat root growth for a better allocation of P to shoots with positive consequences on nutrients uptake and photosynthetic performance.

## Materials and methods

### Experimental set-up and plant growth conditions

A three-month greenhouse experiment was carried out at the Agriculture Innovation and Technology Transfer Center (AITTC) at UM6P in Benguerir, Morocco. The durum wheat [*Triticum turgidum* subsp. *durum* (Desf.) Husn.] variety ´Karim was used in this study and is known as one of the most important and cultivated wheat varieties in Morocco. The wheat plants were grown under greenhouse conditions in plastic cylinders (9.5 cm in diameter and 30 cm in length) containing 2.5 kg of growth substrate composed of a sieved P-deficient (6 ppm of available P measured according to the Olsen method) soil (collected from the AITTC experimental farm), nutrient-free peat, and sand at a ratio of 2:0.5:0.5 (V:V:V), respectively. Wheat seeds were surface disinfected by successive immersion in ethanol (70%, 1 min) and sodium hypochlorite (6%, 5 min), followed by several washes using sterile distilled water. The disinfected seeds were sown at a rate of eight seeds per cylinder with only four plantlets kept after germination. The application of PolyPs fertilizers was done using three PolyPs (PolyB, PolyA and PolyC; three linear PolyPs with short, middle, and long chain length, respectively) and one orthophosphate (OrthoP) according to a recent study by Chtouki et al. [35] conducted on chickpea grown under conditions similar to our study’s conditions. The average daily light intensity was approximatively PAR 280 µmol m^−2^ s^−1^. The four P fertilizers were applied at three doses; 30, 60, and 90 kg P ha^–1^ (namely D30, D60, and D90), respectively. The amounts of N and K nutrients supplied within different P sources were balanced in the Hoagland’s solution [36] for all treatments to a final rate of 180 and 80 kg ha^–1^ for N and K, respectively. Moreover, micronutrients were supplied by irrigation once a week with an NPK-free Hoagland’s solution. All nutrients were supplied in a water solution, including P fertilizers. The negative control pots, P0, received all nutrients except P. The experiment was structured following a randomized complete block design with eight replicates (consisting of 8 cylinders containing 4 plants each) per treatment. During this experiment, the soil moisture was kept constant at 60% of water‐holding capacity measured according to Awlia et al. [37].

### In-situ measured parameters

During 90 days of wheat growth coinciding with the heading stage (Zadok's scale: Z68-Z72), both chlorophyll content index (CCI) and chlorophyll fluorescence were measured *in-situ*. The chlorophyll content index is a non-destructive indicator of chlorophyll content. The CCI was estimated using a portable chlorophyll-meter (Chlorophyll Content Meter, model CL-01, Hansatech Instruments). The CCI values were determined based on dual wavelengths (620 nm and 940 nm) of the spectral absorbance and the results were expressed as a chlorophyll index [38].

Measurement of chlorophyll ‘*a*’ fluorescence was conducted using a portable fluorometer (plant efficiency analyzer, Hansatech Instruments Ltd). Before the *in-situ* measurement, leaves were clipped with black leaf clips for at least 15 min in dark conditions as described by Dewez et al. [39]. The maximum quantum yield (Fv/Fm), the absorption flux per reaction center (ABS/RC) and the performance index (PI) were used in this study as chlorophyll fluorescence derived parameters. The differential curves were obtained by the subtraction of the curve of samples from unfertilized plants (P0) minus the curve of samples from plants that received different PolyPs fertilizers (PolyA, PolyB, PolyC, and OrthoP) [40].

### Plant harvesting and post-harvest analyses

Shoots, roots, and rhizosphere soils were harvested independently after 90 days of growth. Samples of shoots and leaves were stored at − 20 °C for biochemical analyses (e.g., nutrient contents, chlorophyll content and Pi content). The roots were carefully washed until they were free of soil particles and stored in zip-lock bags at − 20 °C for further analyses of morphological root traits, root Pi content, and acid phosphatase (APase) activity. After these measurements, the roots and shoots were dried at 80 °C for 72 h and the shoot (SDW) and root (RDW) dry weight was measured and used to determine mineral (N, P and K) contents. In addition, rhizosphere soil (soil that adheres tightly to the roots) was obtained by gently shaking roots before it was collected in sterile bags and stored at − 20 °C to determine APase activity.

### Measurement of root morphological traits

Root morphological traits, mainly total root length (RL), root surface area (RSA), root volume (RV), and root diameter (RD), were measured using WINRHIZO software (Regent Instruments Inc., QC, Canada). Roots were spread out on a Plexiglas tray filled with water to a depth of 1–2 cm. The tray was placed on a flatbed scanner and imaged at a resolution of 300 dpi with an Epson Expression 836 L scanning system. The obtained images were analyzed with WinRHIZO™ for a quantitative measurement of root traits. Additional root parameters were calculated, notably 1) specific root length (SRL) by dividing root length by root dry mass and 2) root length density (RLD) by dividing root length by root volume.

### Determination of shoot nutrients (N, P, and K) content

After drying shoots at 80 °C for 3 days, the biomass was weighed, and they were finely ground for total N, P, and K content analyses. Wheat shoot powder was digested using nitric acid and analyzed for P and K contents using Inductively Coupled Plasma Optical Emission Spectrometry (Agilent 5110 ICP-OES, USA). The shoot total N content was determined by the Kjeldahl method (KjelMaster K-375, Netherlands).

### Determination of Pi content in shoots and roots

Shoots and roots (aliquots of 100 mg fresh weight) were ground in cold sodium acetate buffer (0.2 M, pH 5.6). After centrifugation (12,000 × *g* at 4 °C for 10 min), an aliquot of the supernatant (50 μL) was used for quantification of inorganic P (Pi). Shoot and root Pi content was determined spectrophotometrically at 880 nm as described by Sun et al. [41]. The root P acquisition efficiency (RPAE mg P g^–1^ RDW) was defined as the amount of P taken up per unit of root biomass according to Elhaissoufi et al. [42]. This ratio reflects the capacity of the root to acquire P from the soil solution.

### Determination of acid phosphatase activity in roots and rhizosphere soils

Root APase activity was measured according to Bargaz et al. [43]. *P*-nitrophenyl phosphate (*p*NPP) was used as a substrate and the enzymatic unit was defined as the amount that catalyzes the hydrolysis of 1 µmol *p*NPP per min per gram of root fresh weight.

Rhizosphere soil APase activity was measured by the addition of 1 g of fresh soil to *p*-nitrophenyl phosphate (*p*-NPP) (10 mM) and acetate buffer (0.2 M). The homogenate was incubated for 1 h at 37 °C. After incubation, APase activity (μmol *p*-NPP h^–1^ g^–1^) was determined spectrophotometrically at 405 nm as described by Bargaz et al*.* [43].

### Determination of leaf chlorophyll content

Total chlorophyll concentration was measured according to Elhaissoufi et al. [42]. An aliquot of 100 mg of fresh leaf tissue was ground in 5 mL of acetone (80%, v/v). After centrifugation, the supernatant was used to measure the optical density at 645 nm (OD663) and 663 (OD645) nm using a spectrometer. Then, the total chlorophyll content was determined using the following formula:

Total chlorophyll content = 8.02 * (OD663) + 20.20 * (OD645).

### Statistical analysis

Statistical analysis of data was performed using IBM® SPSS® software V. 20. Two-way analysis of variance was used to assess the effects of different PolyPs fertilizers and P doses on the wheat above- and below-ground parameters. This analysis was followed by a Tukey’s HSD test to compare the different treatments, and all effects were considered significant at *p* < *0.05*. Pearson correlations were run and used to assess the relationships between below- (morphophysiological root traits, RDW, root Pi) and above-ground parameters (P uptake, photosynthesis related parameters). Principal component analysis (PCA) was performed using Minitab software (version 21.1.0) to visualize the interrelationships among the measured traits in each treatment [42].

## Results

### Effects of PolyPs on wheat root traits

Wheat root trait measurements under PolyPs and OrthoP fertilizers indicated that root morphology was responsive to the P source (Table 1). Specifically, RL, RSA, and RV under PolyPs (PolyA, PolyB, and PolyC) application at D60 were significantly higher compared to both OrthoP-fertilized and unfertilized plants. For instance, PolyC, B, and A significantly increased RL by 130, 42, and 54%, RSA by 60, 40, and 54%, and RV by 63, 53, and 66% unfertilized plants, with RL being the most responsive trait to increasing P dose. The application of PolyC and OrthoP at D30 induced shorter roots compared to D60 and D90, whereas higher RL was observed under PolyA and PolyB at D30. However, RD was significantly lower in response to all PolyP, specifically at D60. In addition, no significant difference was noted between root dry weight under different treatments, with slight increase in RDW of plants fertilized with OrthoP and PolyA.

**Table 1:** Variation in morphological root traits and root biomass of wheat under application of PolyPs and OrthoP at three P doses

P fertilizers	Doses (kg P/ha)	RDW (g)	RL (m)	RSA (cm^2^)	RD (mm)	RV (cm^3^)	RLD (cm.cm^−3^)	SRL (m g^−1^)
**PolyC**	30	2.92 ^abc^	2.40 ^cde^	173.2 ^de^	0.24 ^gh^	1.04 ^ef^	2.33 ^ab^	0.84 ^cde^
	60	2.57 ^bc^	3.79 ^a^	227.78 ^bc^	0.23 h	1.45 ^bc^	2.61 ^a^	1.51 ^a^
	90	2.44 ^c^	3.09 ^bc^	249.74 ^b^	0.24 ^gh^	1.43 ^bc^	2.17 ^bc^	1.31 ^ab^
**PolyB**	30	2.64 ^bc^	3.43 ^ab^	243.04 ^b^	0.24 ^fgh^	1.57 ^b^	2.18 ^bc^	1.3 ^ab^
	60	2.57 ^bc^	2.35 ^de^	198.9 cd	0.26 ^bcd^	1.36 ^bcd^	1.74 ^efg^	0.94 cd
	90	2.82 ^abc^	2.65 cd	241.16 ^b^	0.27 ^abc^	1.45 ^bc^	1.84 ^def^	0.95 cd
**PolyA**	30	3.56 ^a^	3.48 ^ab^	326.05 ^a^	0.26 ^c−f^	1.85 ^a^	1.87 ^cde^	0.99 ^bcd^
	60	3.28 ^abc^	2.54 cd	218.31 ^bc^	0.26 ^b−e^	1.48 ^b^	1.72 ^efg^	0.78 ^de^
	90	3.34 ^ab^	2.39 ^c−e^	239.27 ^b^	0.29 ^a^	1.57 ^b^	1.51 fg	0.72 ^de^
**OrthoP**	30	3.41 ^ab^	2.57 cd	170.96 ^de^	0.27 ^bcd^	1.21 ^cde^	2.12 ^bcd^	0.82 ^cde^
	60	3.16 ^abc^	1.71 ^ef^	143.61 ^e^	0.28 ^ab^	1.16 ^de^	1.48 g	0.57 ^e^
	90	3.01 ^abc^	3.43 ^ab^	225.22 ^bc^	0.25 ^e–h^	1.43 ^bc^	2.39 ^ab^	1.14 ^bc^
**P0**		3.01 ^abc^	1.65 ^f^	141.98 ^e^	0.25 ^d−g^	0.89 ^f^	1.82 ^def^	0.56 ^e^
P		***	***	***	***	***	***	***
D		ns	***	***	***	ns	***	Ns
P * D		ns	***	***	***	***	***	***

Means (*n* = 8) that do not share the same letters in the column differ significantly according to Tukey's test (*p* < 0.05). Asterisks indicate significant differences between P-fertilizers (P), P doses (D) and their interactions (P*D) (ns. not significant; * *p* < 0.05; **p < 0.01; ***P < 0.001)

Furthermore, specific root traits; SRL and RLD, were significantly enhanced in response to PolyPs fertilizers, especially PolyC (Table 1). At D60, both RLD and SRL increased by 43 and 170% under PolyC, 18 and 65% under PolyB, 16 and 37% under PolyA application compared to the unfertilized plants.

### Effects of PolyPs on nutrient (N, P, and K) uptake

The application of PolyPs and OrthoP at D30, D60, and D90 improved the SDW of durum wheat compared to unfertilized plants that showed the lowest shoot dry weight (Table 2). The application of PolyC, B, and A applied at D90 increased SDW by 19, 33, and 15%, respectively. In addition, all P fertilizers significantly increased shoot nutrient (N, P, K) contents compared to unfertilized plants (Table 2). The application of PolyC, B, and A at D60 enhanced total shoot N content by 276, 305, and 282%, respectively, compared to the unfertilized treatment. Although no significant difference was found in total P and K contents between the four P-fertilizers, application of PolyC, B, and A at D60 increased K content by 124, 129, and 115%, respectively, compared to unfertilized plants. Similarly, total P content was significantly increased by 327% under PolyC compared to unfertilized plants.

**Table 2:** Variation in shoot nutrient (N, P K; mg/total SDW/cylinder) contents of wheat under application of PolyPs and OrthoP at three P doses

P fertilizers	Doses (kg P/ha)	SDW	N	P	K
**PolyC**	30	9.8 ^ab^	159.1 ^a−d^	19.6 ^ab^	180.4 ^abc^
	60	11.2 ^a^	204.2 ^ab^	33.3 ^a^	230.4 ^ab^
	90	9.9 ^ab^	244.3 ^a^	32.7 ^a^	205.3 ^abc^
**PolyB**	30	10.8 ^a^	220.1 ^ab^	19.7 ^ab^	221.4 ^ab^
	60	10.2 ^ab^	219.7 ^ab^	26.8 ^a^	235.3 ^ab^
	90	11 ^a^	258.2 ^a^	32.3 ^a^	261.2 ^a^
**PolyA**	30	10.4 ^ab^	200.0 ^ab^	23.8 ^ab^	200.8 ^abc^
	60	10.5 ^ab^	207.5 ^ab^	26.9 ^a^	220.8 ^ab^
	90	9.5 ^ab^	106.5 cd	17.7 ^ab^	137.2 ^bc^
**OrthoP**	30	10.8 ^a^	169.0 ^abc^	17.4 ^ab^	196.8 ^abc^
	60	10.8 ^a^	128.1 ^bcd^	27.1 ^a^	206.4 ^abc^
	90	8.3 ^ab^	122.3 ^bcd^	30.7 ^a^	189.5 ^abc^
**P0**		6.7 ^b^	54.3 ^d^	7.8 ^b^	102.7 ^c^

Means (*n* = 4) that do not share the same letters in the column differ significantly according to Tukey's test (*p* < 0.05). Asterisks indicate significant differences between P-fertilizers (P), P doses (D) and their interactions (P*D) (ns. not significant; *p < 0.05; **p < 0.01; ***p < 0.001)

### Effects of PolyPs on wheat Pi content and phosphatase activities

**Fig. 1:**
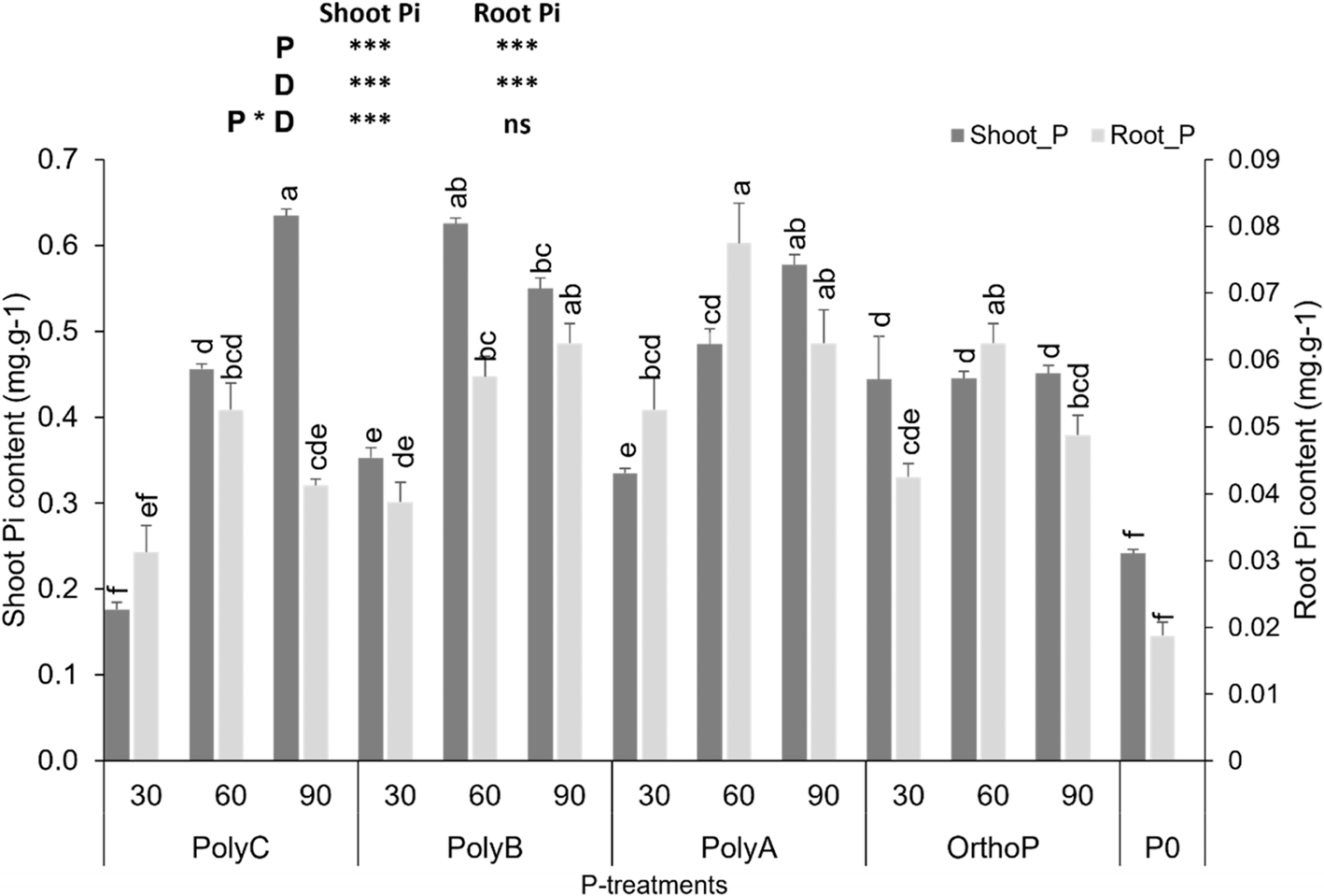
Variation in shoot and root Pi contents of wheat under PolyPs and OrthoP application at three P doses. Data are mean values ± SD (*n* = 8), Different lowercase letters above the bars indicate significant differences (*p* < 0.05) according to Tukey’s test. Asterisks indicate significant differences between P-fertilizers (P), P doses (D) and their interactions (P*D) (ns. not significant; *p < 0.05; **p < 0.01; ***p < 0.001)

Wheat plants fertilized with the three PolyPs (especially at D60 and D90) showed significant increases in shoot Pi content compared to other treatments (Fig. 1). For instance, the application of PolyC, B, and A at D90 enhanced shoot Pi by 88, 159, and 101% compared to the unfertilized plants. However, the application of OrthoP at D30 increased shoot Pi content compared to PolyC (151%), PolyB (26%), and PolyA (32%) all at the same dose. In terms of P doses, shoot Pi was clearly enhanced with the three PolyPs at D60 and D90 when compared to D30, where shoot Pi was significantly low. Conversely, root Pi content showed no significant differences regardless of the type of P fertilizer. In comparison to unfertilized plants, application of all P fertilizers significantly improved root Pi content.

**Fig. 2:**
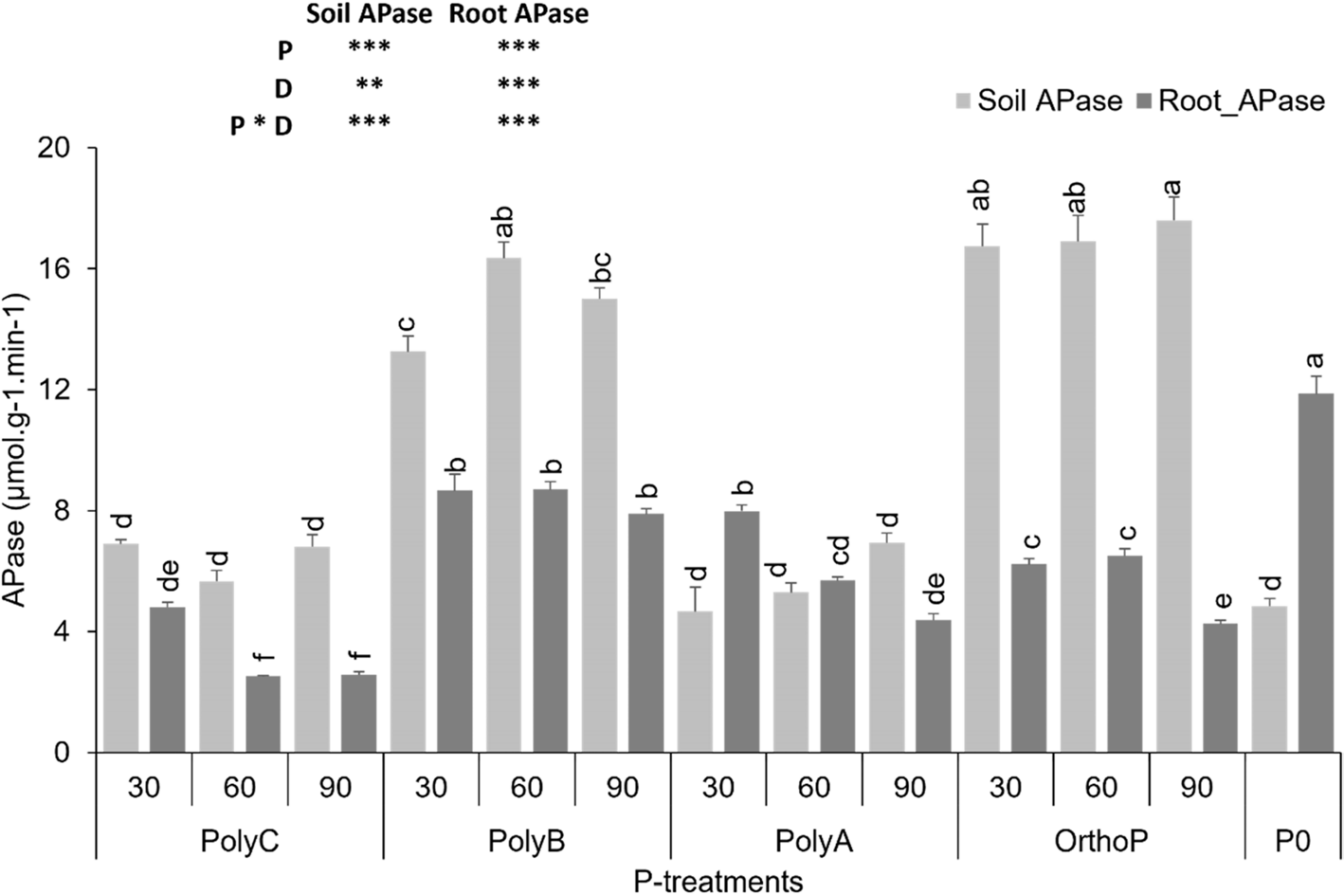
Variation in soil and root acid phosphatases activity under PolyPs application at three P doses. Data are mean values ± SD (*n* = 8), Different lowercase letters above the bars indicate significant differences (*p* < 0.05) according to Tukey’s test. Asterisks indicate significant differences between P-fertilizers (P), P doses (D) and their interactions (P*D) (ns. not significant; *p < 0.05; **p < 0.01; ***p < 0.001)

Results in Fig. 2 show that root APase activity significantly increased in both PolyB- and PolyA-fertilized plants when compared to plants fertilized with PolyC or OrthoP. Root APase activity increased by 33 and 85% in response to application of PolyB at D60 and D90, respectively, compared to plants fertilized with OrthoP at the same doses. It is worth noting that unfertilized plants exhibited the highest root APase activity, which is evident due to their P-deficiency status. In addition, the application of OrthoP and PolyB, with no significant differences between the three doses, significantly increased APase activity in rhizosphere soil in comparison to other treatments. Therefore, soil APase activity increased 3 times under OrthoP and PolyB compared to unfertilized soil.

### Effects of PolyPs on P uptake and root acquisition efficiency

**Fig. 3:**
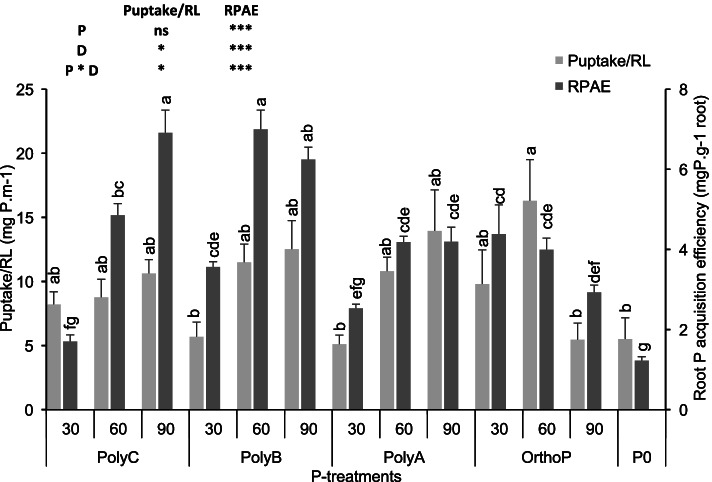
Variation in P uptake and root P acquisition efficiency of wheat under application of PolyPs and OrthoP at three P doses. Data are mean values ± SD (*n* = 8), Different lowercase letters above the bars indicate significant differences (*p* < 0.05) according to Tukey’s test. Asterisks indicate significant differences between P-fertilizers (P), P doses (D) and their interactions (P*D) (ns. not significant; *p < 0.05; **p < 0.01; ***p < 0.001)

Root P acquisition efficiency (RPAE) improved with increasing P doses of the three PolyP. However, RPAE was decreased with increased OrthoP dose (Fig. 3). For instance, the application of PolyC and PolyB at D60 significantly increased RPAE by 296 and 471%, respectively, compared unfertilized treatment. Similarly, the P uptake per unit of RL was improved under PolyC, B, and A at D60 and D90 compared to other treatments. The P uptake per RL was increased by increased by 59, 109, and 96% under the application of PolyC, B and A compared to the unfertilized treatments.

### Relationships between morpho-physiological root traits and P uptake

In this study, strong relationships were found between morphophysiological root traits and P uptake of wheat under PolyPs application compared to OrthoP application. Except for RV and root APase activity under PolyB, significant correlations were found between shoot Pi content under PolyB and PolyA application and root traits such as RL (r =  − 0.67^**^, r =  − 0.58^**^, respectively), RV (r =  − 0.35, r =  − 0.42^*^, respectively), RSA (r =  − 0.46^*^, r =  − 0.61^**^, respectively), RLD (r =  − 0.70^**^, r =  − 0.61^**^, respectively), SRL (r =  − 0.57^**^, r =  − 0.49^**^, respectively), and root APase activity (r =  − 0.07, r =  − 0.89^**^, respectively). This may indicate that PolyPs induce specific root morphological changes responsible for efficient P acquisition. However, significant positive correlations under the application of PolyB and PolyA were found between shoot Pi and both RD (r = 0.78^**^, r = 0.81^**^, respectively) and soil APase activity (r = 0.69^**^, r = 0.56^**^, respectively). In addition, positive correlations were found between the above-stated parameters under the application of PolyC and OrthoP.

In addition, shoot Pi was significantly positively correlated with both soil APase activity and P-uptake/RL in response to PolyB and PolyA application, whereas no significant correlation between these parameters was noted under OrthoP application. Another positive correlation was found between morphological traits such as RL (r = 0.75^**^), RSA (r = 0.71^**^), RV (r = 0.58^**^), RLD (r = 0.77^**^), and SRL (r = 0.63^**^) and physiological root traits (APase activity) only under PolyA application.

### Photosynthesis performance of wheat under PolyPs application

**Fig. 4:**
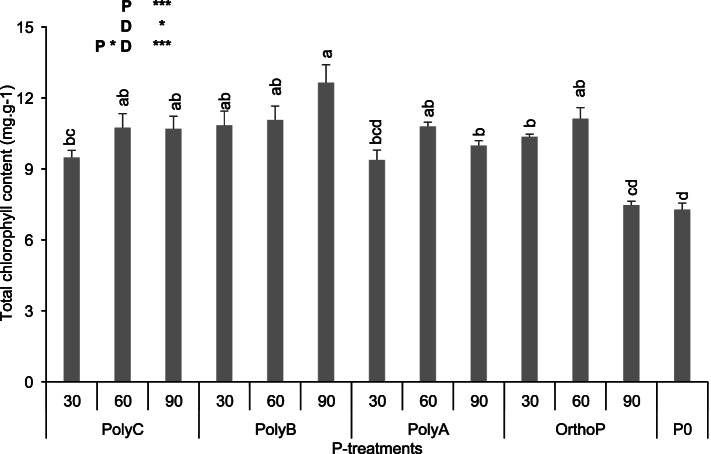
Variation in total chlorophyll content of wheat under application of PolyPs and OrthoP at three P doses. Data are mean values ± SD (*n* = 8), Different lowercase letters above the bars indicate significant differences (*p* < 0.05) according to Tukey’s test. Asterisks indicate significant differences between P-fertilizers (P), P doses (D) and their interactions (P*D) (ns. not significant; *p < 0.05; **p < 0.01; ***p < 0.001)

Except for OrthoP at D90, Chlorophyll content (CCI and total chlorophyll) increased significantly under all P fertilizer regimes, with differential effects regarding the P fertilizer type, compared to unfertilized plants (Fig. 4, Fig. S1). For instance, CCI slightly enhanced under PolyC (11 and 13%) and OrthoP (7 and 8%) at D60 compared to PolyA and PolyB. Additionally, total chlorophyll content increased by 46, 73, and 37% under PolyC, B, and A at D90 when compared to unfertilized treatments.

Similarly, the PI under PolyC and PolyB at D60 significantly increased compared to other treatments that showed the lowest PI (Table 3). Similarly, ABS/RC was low in plants fertilized with the three PolyPs at D60 compared to both OrthoP- and unfertilized plants. A significant increase in ABS/RC ratio in unfertilized plants may indicate an increase in inactivated reaction centers compared to absorbed photons, which is evident under stressful conditions such as low P availability. However, the potential quantum efficiency of PSII reflected by the Fv/Fm ratio was significantly higher under the application of the four fertilizers (PolyPs and OrthoP) compared to zero P application. Likewise, unfertilized plants had the lowest CCI (two times lower than fertilized plants), total chlorophyll content, and parameters derived from chlorophyll *a* fluorescence, which may be attributed to negative effects of low P availability on the photosynthesis process.

**Table 3:** Variation in chlorophyll fluorescence parameters of wheat under application of PolyPs and OrthoP at three P doses

P fertilizers	Doses (kg P/ha)	Fv/Fm	ABS/RC	PI
PolyC	30	0.82 ^b^	2.23 ^d^	1.17 ^ab^
	60	0.82 ^a^	2.29 cd	1.22 ^a^
	90	0.8 ^a^	2.38 cd	0.76 cd
PolyB	30	0.8 ^ab^	2.43 cd	0.71 ^cde^
	60	0.80 ^a^	2.38 cd	0.89 ^abc^
	90	0.77 ^ab^	2.71 ^ab^	0.52 ^de^
PolyA	30	0.81 ^ab^	2.35 cd	0.93 ^abc^
	60	0.79 ^a^	2.38 cd	0.7 ^cde^
	90	0.8 ^ab^	2.5 ^bc^	0.79 cd
OrthoP	30	0.80 ^a^	2.39 cd	0.89 ^abc^
	60	0.80 ^ab^	2.48 ^bcd^	0.75 ^cde^
	90	0.80 ^a^	2.36 cd	0.86 ^bc^
P0		0.77 ^a^	2.81 ^a^	0.42 ^e^
P		***	***	***
D		**	**	***
P * D		***	*	***

Means (*n* = 8) that do not share the same letters in the column differ significantly according to Tukey's test (*p* < 0.05). Asterisks indicate significant differences between P-fertilizers (P), P doses (D) and their interactions (P*D) (ns. not significant; *p < 0.05; **p < 0.01; ***p < 0.001)

Positive and significant correlations between total chlorophyll content, CCI, Fv/Fm, and PI were found in response to OrthoP application (Table 4) as opposed to ABS/RC that showed negative and significant correlation with CCI and chlorophyll content. However, there are no significant correlations between the parameters derived from the chlorophyll *a* fluorescence and chlorophyll content (Table 4).

**Table 4:** Correlations (Pearson´s correlation) between chlorophyll content and chlorophyll fluorescence-related parameters of wheat under application of PolyPs and OrthoP at three P doses

P fertilizers	Fluorescence Parameters	Chla	Chlb	Chlt	CCI
**PolyC**	Fv/Fm	0.26	-0.33	-0.07	0.70^**^
	ABS/RC	-0.40	0.51^*^	0.11	-0.49^*^
	PI	0.33	-0.47^*^	-0.14	0.77^**^
**PolyB**	Fv/Fm	-0.03	0.10	0.05	0.29
	ABS/RC	0.15	-0.26	-0.07	-0.10
	PI	0.05	0.03	0.07	0.19
**PolyA**	Fv/Fm	-0.26	-0.06	-0.26	-0.33
	ABS/RC	0.09	0.18	0.18	-0.29
	PI	-0.31	-0.15	-0.36	-0.08
**OrthoP**	Fv/Fm	0.57^**^	-0.01	0.45^*^	0.51^*^
	ABS/RC	-0.51^*^	-0.14	-0.47^*^	-0.46^*^
	PI	0.54^**^	0.17	0.51^*^	0.47^*^
**P0**	Fv/Fm	-0.12	-0.26	-0.34	-0.03
	ABS/RC	0.29	-0.77^*^	-0.80^*^	-0.04
	PI	-0.24	0.49	0.49	0.31

Asterisks indicate significant correlation at *p < 0.05; **p < 0.01; ***p < 0.001)

**Fig. 5:**
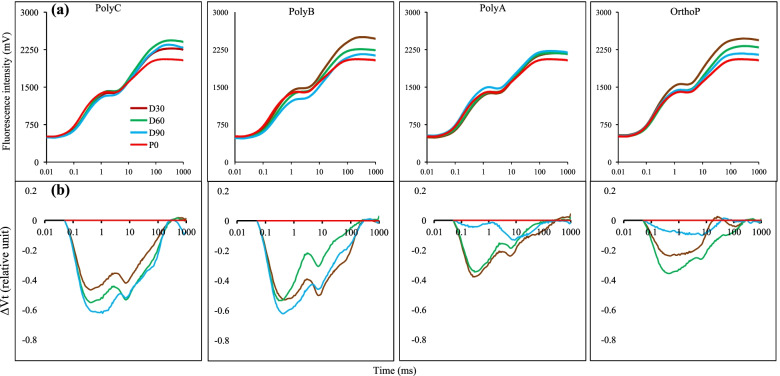
Effects of PolyPs and OrthoP application at three P doses on chlorophyll a fluorescence transient curves **a** and on differential chlorophyll fluorescence curves ΔVt **b** of wheat. Each value represents the mean of eight independent repetitions. The differential curves (ΔVt) were constructed by subtracting the normalized fluorescence values recorded in fertilized plants from those recorded in the unfertilized ones (P0)

Chlorophyll *a* fluorescence transients and differential curves (ΔVt) were used to evaluate the effect of PolyPs on photosynthesis light reactions which indirectly reflects the photosynthesis performance (Fig. 5). The chlorophyll *a* fluorescence curves were recorded in dark-adapted leaves and are shown in Fig. 5A. At the initial chlorophyll fluorescence level (FO), no significant change was noted between the different P doses regardless of the type of PolyP applied to the plants. In contrast, a clear increase in the maximal chlorophyll fluorescence level (FM) was observed in leaves of fertilized plants compared to unfertilized ones. Visible changes in the shape of the chlorophyll *a* fluorescence transient were also noted at FM level. Obvious differences were noted between the P doses of PolyPs fertilizers when compared to other treatments that showed a similar FM for the three doses. The negative amplitude of the ΔVt bands in plants under the application of PolyPs and OrthoP at different doses (Fig. 5B) suggests a positive impact of P application on photosynthesis efficiency.

## Discussion

The present study contributes to enrich the available knowledge on wheat growth performance under PolyPs application, specifically traits related to root functioning, P-acquisition, and above-ground physiology with a focus on P use efficiency and photosynthetic activity. This study revealed key interconnections between root traits, physiological and morphological aspects, P-acquisition, and above-ground physiological changes related to photosynthesis efficiency (estimated by chlorophyll fluorescence measurements) and uptake of nutrients. Findings of the present study describe, for the first time, the role of PolyPs in modulating morphological and physiological traits in both root and rhizosphere, which resulted in a better use of PolyPs and a higher P uptake of durum wheat where both fundamental and applied knowledge remains scarce to date.

### Polyphosphate fertilizers can stimulate wheat root system development and functioning

Our findings showed that the application of different PolyPs fertilizers significantly influenced both the morphology and physiology of roots compared to other treatments (Table 1). For instance, PolyA application enhanced RDW compared to other PolyPs fertilizers (PolyB and PolyC). This was in agreement with findings by Gao et al. [15] that greenhouse-grown maize plants fertilized with ammonium PolyP enhanced plant dry biomass, especially root dry biomass. The same study reported a significant correlation (r = 0.91) between maize total dry biomass and P uptake under ammonium PolyP application. Similarly, findings by Torres-Dorante et al. [13] showed that application of PolyPs (pyrophosphate and trimetaphosphate) stimulated maize root growth through increasing root length compared to sodium OrthoP application. These findings were consistent with our results that application of PolyPs (PolyA, B, and C), notably at D60, enhanced root traits such as SRL (increased by 16, 17 and 76% compared to the unfertilized plants) which is an important trait that facilitates the exploration of a large surface area with a relatively small investment in root biomass. This finding might be attributed to the role of PolyPs in modulating wheat root growth for efficient P acquisition. In line with nutrient root uptake efficiency, higher SRL is recognized as acquisitive root trait expression that is tightly coupled with increased P-acquisition efficiency in various crop species, including wheat, under contrasting regimes of P [32, 44]. This is consistent with our results indicating that root system of wheat fertilized with PolyPs at D60 exhibited higher RV and RSA can be a result of enhancing soil P exploration. These results were found under PolyPs application at D60 and this is presumably considered to be the adequate PolyP dose for durum wheat growth as it allows adequate root growth performance under the tested experimental conditions. Therefore, high P uptake under PolyPs application at D60 could be linked to the large root system (high RV and RSA), which would have allowed progressive hydrolysis of PolyPs through rhizosphere acidification (e.g., secretion of protons and organic acids) and secretion of P-hydrolyzing enzymes, assuming these two mechanisms are involved in PolyPs hydrolysis [14, 20, 45]. Comparable root responses under adequate P application (200 μmol/L KH_2_PO_4_) were reported by Wang et al. [46] in different wheat genotypes (grown under hydroponic conditions) exhibiting high RL and RSA compared to plants under low P application.

One of the most important root traits contributing to enhanced P absorption is RLD, which is positively correlated with P use efficiency in wheat grown under field conditions, especially under OrthoP fertilizer application compared to unfertilized wheat [31, 47]. These findings are consistent with our results indicating that PolyPs plausibly “directly or indirectly” modulated specific functional and structural root traits that significantly contributed to enhancing wheat P acquisition and above-ground performance. Additionally, other studies found that RLD of wheat and soybean significantly increased with increasing P rate and this was significantly correlated with grain yield under field conditions [48–50]. Furthermore, it is worth noting that RL was responsive to increasing P dose, especially under the application of PolyA and PolyB. Similarly, RL also increased with increasing P dose under PolyC and OrthoP fertilizers, which is consistent with the finding by Shen et al. [51] that RL of wheat grown under greenhouse conditions increased with increasing P (KH_2_PO_4_) application. Likewise, Torres-Dorante et al. [13] reported that sodium PolyP increased maize RL compared to di-sodium hydrogen OrthoP application. Unlike other root traits, RD was significantly lower under PolyC, B, and A at D60 compared to other treatments. It has been reported that many cereals (maize, wheat, etc.) are characterized by a smaller RD than legume crops (Lupin, faba bean, etc.), given its role in P acquisition allowing high absorptive capacity [51, 52]. Our findings on RD variations agree with the general observation of other studies, supporting the fact that RD is an important morphological root trait involved in P acquisition efficiency, especially in cereal crops according to available knowledge [32, 51–55].

### Polyphosphate application enhanced P acquisition in durum wheat

The present study demonstrated that PolyPs fertilizers, especially PolyB and PolyC at D60 and D90 significantly improved shoot Pi content, root P acquisition efficiency, and P uptake per unit of root length (Fig. 1, 3). Improved translocation of P to shoots under all PolyPs fertilizers applications is consistent with the few available studies reporting enhanced P uptake under ammonium PolyP compared to OrthoP fertilizer application, such as monoammonium P, diammonium P, and triple super P [9, 19, 56]. This has also been confirmed recently by Gao et al. [15] who found that application of ammonium PolyP (60 kg P ha^−1^) significantly improved P uptake (shoot and root P) of maize in pot experiments. Improvement of maize P uptake under ammonium PolyP may be due to progressive enhancement of soil available P from this PolyP [13, 15]. In addition, a greenhouse experiment conducted by McBeath et al. [9] found a significant increase in wheat shoot biomass and P tissue concentration under ammonium PolyP application compared to monoammonium P, which is consistent with our findings for PolyC, B, and A. Another study by McBeath et al. [19] confirmed that shoot P content significantly (r = 0.97) correlated with wheat shoot biomass in response to application of ammonium PolyP fertilizer compared to triple superphosphate. Furthermore, our findings demonstrate a significant improvement of root P acquisition efficiency by 5, 75, and 22% under the application of PolyA, PolyB, and PolyC, specifically at D60. This presumably indicates that PolyPs application promotes investment in root mass, along with less P accumulation in roots (expressed in lower root Pi content compared to shoots), which helps to allocate the required amounts of soil nutrients, especially P, to shoots and thus contributes to sustaining physiological processes and production of grain yield. Similarly, P uptake per unit of RL was enhanced in response to all P-fertilizers applied at D60 and D90 compared to unfertilized wheat plants. This trait was reported to increase soil available P and contribute to a better P acquisition efficiency in many crops including wheat, pea, and lupin [57].

In relation to P acquisition, plants adapt several mechanisms to cope with low P availability including modulation of their root growth (tradeoffs and interactions between morphological, anatomical, and physiological root traits), recruitment of rhizosphere microbial communities (e.g. specific arbuscular mycorrhizal fungi and plant growth promoting bacteria) and alteration of the rhizosphere biology and chemistry linked to P availability [31, 58–62]. The exudation of organic acids and P-hydrolyzing enzymes (phosphatases and phytases) by plant roots and rhizosphere microbes are among the main physiological mechanisms involved in enhancing root P acquisition. In connection with our study, phosphatases secreted by roots are also important enzymes involved in PolyPs hydrolysis [32, 45, 63–65]. In this regard, our study indicates that APase activities in root and rhizosphere soil significantly increased under PolyB application, particularly at D60 and D90 compared to unfertilized treatment (Fig. 2). Rhizosphere soil APase significantly correlated with shoot Pi (r = 0.69^**^, r = 0.56^**^) under the application of PolyA and PolyB. This finding concurs with previous studies highlighting the key role of P-hydrolyzing enzymes in the progressive hydrolysis of PolyPs and P allocation to shoots [14, 20, 34, 66]. Secretion of these enzymes into the rhizosphere soil has been described as an important enzymatic pathway for PolyPs hydrolysis in soils [45, 67, 68], which is significantly influenced by root activity. In the present study, morphological root traits such as RL and RSA significantly correlated with root APase activity under PolyC (r =  − 0.71**, r =  − 0.85**) and PolyA (r = 0.75**, r = 0.71**). These findings suggest that PolyPs application may impact the root trade-off between morphological and physiological traits related to P acquisition efficiency. It can moreover be suggested that PolyP type impacts the root trade-off differently between morphological and physiological traits related to P acquisition efficiency. In addition, these various responses could be attributed to the impact of each PolyP on rhizosphere microbial activities given the key role of microorganisms in PolyPs hydrolysis.

### Wheat P acquisition and root traits are interconnected under PolyPs application

Several studies have found that P-uptake is strongly influenced by trade-offs between diverse root functional traits related to P-acquisition, and which can vary considerably within or between plant species [32, 52, 69–71]. However, little is known about how these trade-offs and coordination between root traits cooperate to enhance P acquisition in response to P availability and applied P-types (PolyP and OrthoP). In this present study, significant correlations between root traits and P acquisition can explain the improvement of overall wheat growth under PolyPs application. For instance, PolyB- and PolyA-fertilized plants expressed significant correlations between shoot Pi content and RL, RV, RSA, RLD, SRL, and root APase activity (Table S1). In this regard, Honvault et al. [72] found that shoot P concentration negatively correlated with morphological root traits such as RSA (r =  − 0.30). This latter study explained this correlation by the differential expression of root traits involved in P acquisition depending on their carbon cost for plants and P availability status in the rhizosphere. The plants may in fact express one or several traits depending on their carbon cost and P availability changes in the environment surrounding roots [32, 52, 63, 73, 74]. However, PolyC application resulted in positive correlations between the above-mentioned morphological root traits and shoot Pi. These correlations presumably indicate that functional root trait trade-offs and interactions involved in P uptake were differently impacted according to the PolyP type. In this study, positive correlations were noted between root physiological (APase activity) and morphological (RL, RSA, RV, RLD, and SRL) traits under PolyA, while negative correlations between these parameters were noted under PolyC application. These contrasting effects seem to be PolyP-type dependent which is partly explained by the difference in PolyP chain length. This difference in chain length, among other potential properties, can significantly affect their hydrolysis and consequently P availability in the rhizosphere. For instance, a study conducted by Dick and Tabatabai [45] showed that phosphatases exuded by corn roots and their associated microbes can hydrolyze different PolyPs to a lesser extent for long chain length PolyPs (P35 and P65) and cyclic PolyPs that appeared to be less responsive to enzymatic hydrolysis. These findings are consistent with previous studies reporting that P-acquisition efficiency may be achieved through a complex of interactions (expressed by positive or negative correlations) between root morpho-physiological traits [32, 52, 65]. More specifically, Wen et al. [65] reported that increased maize shoot P content was accompanied by a decrease in morphological root traits (e.g., RL and RSA) and an enhancement of physiological root traits (e.g., APase activity and concentration of carboxylates such as malate, citrate, and succinate). Nevertheless, the interactions between root traits that directly govern P acquisition efficiency are still poorly understood given the diversity of quantitative trait locus controlling P acquisition between and within plant species [65, 69, 72]. Results from PCA demonstrate that improved root traits do only not influence P uptake, but also improve nutrients uptake and physiological parameters under the PolyPs application. The first two principal components explained together 56.33% of the total variation. This analysis showed three main clusters of traits can be visualized: a first clustering morphological root traits (RL, RSA and RV), specific root traits (RLD and SRL) and fluorescence parameters (Fv/Fm and PI) and N content showing strong correlations in responses to PolyB at D30 and PolyC at D60; a second group comprising chlorophyll content (CCI and total chlorophyll), nutrients contents (P and K), P acquisition efficiency traits (RPAE and P uptake/RL) that significantly correlates in response to PolyB (D60 and D90) and PolyA at D60; and a third group including ABS/RC, root APase and RDW that seems to be more influenced by OrthoP application (Fig. S2).

### Polyphosphate fertilizers improved photosynthetic performance and nutrient uptake

Apart from the direct effects of PolyP on P uptake, their application also improved wheat above-ground parameters, notably SDW and nutrients (N, P and K) uptake and photosynthesis-linked parameters (Table 2, Fig. 4). In line with that, it has been reported that P deficiency alters the photosynthesis process through significant alteration of NADPH regeneration, which reduces the quantum yield and electron transport efficiency [75]. In this study, plants fertilized with both PolyPs (to a lesser extent for PolyA and PolyC) and OrthoP exhibited higher chlorophyll content compared to unfertilized plants, which is consistent with previous findings that efficient P acquisition significantly increases the net rate of photosynthesis [76–78] that is directly linked to crop growth and yield. In addition, chlorophyll fluorescence derived parameters such as Fv/Fm and PI were improved in response to P application (PolyPs and OrthoP) compared to unfertilized plants, where the lowest PI and Fv/Fm were recorded. Similarly, the unfertilized plants showed higher ABS/RC compared to P-fertilized (with both PolyPs and OrthoP) plants, indicating that P deficiency negatively affects the electron transport chain and may induce the inactivation of some PSII reactional centers. The analysis of chlorophyll *a* fluorescence transients showed that PolyPs fertilizers not only improved P acquisition but also photosynthesis efficiency through ensuring optimal functioning of photosynthetic apparatus. Based on many studies conducted on the role of P in photosynthesis performance, it has been clearly reported that P deficiency significantly alters the electron transport chain, net photosynthetic rate, and maximum quantum efficiency of PSII along with a reduction in the activity of some key enzymes in the Calvin cycle [79, 80]. The chlorophyll fluorescence transients curves indicate that P-fertilizers at different doses improved the photosynthesis apparatus of wheat, with slight differences between PolyPs and OrthoP (Fig. 5). The decline in FM could partly be related to a reduction in chlorophyll *a* concentration, plausibly indicating a response to stressful conditions [81, 82]. It has been reported that P-deficiency triggers photooxidative stress responses leading to chlorophyll loss and a decrease in quantum yield of PSII [83]. The negative k-band (~ 300 µs) peaks in the curves can be explained by faster electron transport in plants in response to PolyPs and OrthoP application compared to unfertilized plants (Fig. 5). This result indicates that electron transport from the oxygen evolving complex to the reaction center at the acceptor site of PSII was maintained in response to different P fertilizers (compared to unfertilized plants) with slight improvements under PolyC and PolyB (higher k-band amplitude), which is in line with previous findings [39, 84]. In addition, a negative K band was observed in leaves of chickpea plants under PolyP application, which indicates that electron transport to the donor side of PSII was improved under PolyP application [35]. It is worth mentioning that chlorophyll fluorescence analysis provides strong evidence of photosynthetic efficiency and could be a good non-destructive tool to predict actual crop P status and help to determine the adequate P application allowing the achievement of maximum yield [82, 85]. Additionally, the observed improvement of photosynthesis could be due to the significant improvement in shoot N content after all PolyPs applications at D60, which agrees with findings by Gvozdevaite et al. [86] who reported that the functioning of photosynthesis machinery depended, among other elements, on N content translocated to shoots.

## Conclusions

Given the lack of knowledge on the mechanisms of action of PolyPs and their effects on P acquisition and growth performance, use of PolyPs in intensive farming systems is still limited. These fertilizers can be considered efficient multifeatured P fertilizers having the potential to stimulate plant growth and P acquisition efficiency. Our findings demonstrate that PolyPs (especially PolyB and PolyC) application enhanced P acquisition and improved wheat above-ground performance. Specifically, application of PolyPs (especially PolyB and PolyA) at D60 showed promising results in terms of plant growth and P uptake, suggesting that PolyPs application at D60, under the current study conditions, improved wheat growth performance, photosynthesis, and P acquisition efficiency. Improvement of P acquisition under PolyPs application could be linked to changes in morphological and physiological root traits. Moreover, obvious enhancement of above-ground parameters, notably nutrient and chlorophyll contents, could be reliable indicators of the advantageous effects of PolyPs on the whole plant growth performance as well as below- and above-ground interconnections. Therefore, the beneficial effects of PolyPs could be further explored by discerning specific patterns of relationships between above- and below-ground parameters through greenhouse and field experiments. Moreover, this study opens a new route of research to investigate specific below-ground mechanisms (functional traits of roots and associated microbes) driving PolyPs hydrolysis in soils, which will help to assess the slow-release properties that PolyPs could exhibit. In this regard, field and greenhouse experiments are needed to accurately determine the importance of below-above-ground interconnections for a better acquisition of P under PolyPs application. Also, understanding the role of rhizosphere soil microbiota in response to PolyPs application is highly recommended, especially P solubilizing/mineralizing microorganisms with high potential for production of organic acids and phosphatases through stimulating specific root physiological traits presumably involved in PolyP use efficiency.

## Supplementary Information

**Additional file 1: Figure S1.**Variation in chlorophyll content index of wheat under application of PolyPs and OrthoP at three P doses. Data are mean values ± SD (*n*=8), Different lowercase letters above the bars indicate significant differences (*p* ≤ 0.05) according to Tukey’s test. Asterisks indicate significant differences between P-fertilizers (P), P doses (D) and theirinteractions (P*D) (ns. not significant; *p < 0.05; **p < 0.01; ***p < 0.001). **Figure S2.** Principal component analysis elaborated based on biomasses, soil acid phosphatases activity, nutrients uptake, morpho-physiological root traits and photosynthesis linked-parameters measured in durum wheat fertilized with PolyPs and OrthoP at three doses. *PolyP, * Polyphosphate; *OrthoP,* orthophosphate; *RDW,* root dry weight; *SDW,* shoot dryweight; *RL,* root length; *RSA,* root surface area; *RV,* root volume, *RD,* rootdiameter; *SRL,* specific root length; *RPAE,* Root P acquisition efficiency; *RLD,* root length density; *APase,* acid phosphatase; *shoot_Pi,* shoot Pi content; *Root_Pi,* Root Pi content; N, P and K: N, P and K content in the shoot; *Chla,* chlorophyll a content; *Chlb,* chlorophyll b content; *Chlt,* total chlorophyllcontent; *CCI,* chlorophyll content index; *Fv/Fm ratio,* quantum efficiency of *PSII,* ABS/RC: the absorption flux per reaction center and *PI,* performance index. **Table S1.** Correlations (Pearson’s correlation) between root morpho-physiological traits and Pi content (shoot and root) of wheat under application of PolyPs and OrthoP at three P doses.

## Data Availability

The datasets supporting the findings of this article are included within the article and its additional files. The raw data used in this study are available from the corresponding author (Adnane Bargaz) on reasonable request.

## Abbreviations

PolyP: Polyphosphate
OrthoP: Orthophosphate
RL: Root length
RSA: Root surface area
RV: Root volume
RD: Root diameter
RDW: Root dry weight
SDW: Shoot dry weight
SRL: Specific root length
RPAE: Root P acquisition efficiency
RLD: Root length density
APase: Acid phosphatase
Chla: Chlorophyll a content
Chlb: Chlorophyll b content
Chlt: Total chlorophyll content
CCI: Chlorophyll content index
Fv/Fm: Reflects the potential quantum efficiency of PSII
ABS/RC: The absorption flux per reaction center
PI: Performance index
