# Supplementary material for: Polyphosphate application influences morpho-physiological root traits involved in P acquisition and durum wheat growth performance
Source: BMC Plant Biol. 2022 Jun 27;22:309. doi: 10.1186/s12870-022-03683-w (PMC9235221; doi:10.1186/s12870-022-03683-w)
Supplement: Supplementary file 1 — Additional file 1: Figure S1.Variation in chlorophyll content index of wheat under application of PolyPs and OrthoP at three P doses. Data are mean values ± SD (n=8), Different lowercase letters above the bars indicate significant differences (p ≤ 0.05) according to Tukey’s test. Asterisks indicate significant differences between P-fertilizers (P), P doses (D) and theirinteractions (P*D) (ns. not significant; *p < 0.05; **p < 0.01; ***p < 0.001). Figure S2. Principal component analysis elaborated based on biomasses, soil acid phosphatases activity, nutrients uptake, morpho-physiological root traits and photosynthesis linked-parameters measured in durum wheat fertilized with PolyPs and OrthoP at three doses. PolyP, Polyphosphate; OrthoP, orthophosphate; RDW, root dry weight; SDW, shoot dryweight; RL, root length; RSA, root surface area; RV, root volume, RD, rootdiameter; SRL, specific root length; RPAE, Root P acquisition efficiency; RLD, root length density; APase, acid phosphatase; shoot_Pi, shoot Pi content; Root_Pi, Root Pi content; N, P and K: N, P and K content in the shoot; Chla, chlorophyll a content; Chlb, chlorophyll b content; Chlt, total chlorophyllcontent; CCI, chlorophyll content index; Fv/Fm ratio, quantum efficiency of PSII, ABS/RC: the absorption flux per reaction center and PI, performance index. Table S1. Correlations (Pearson’s correlation) between root morpho-physiological traits and Pi content (shoot and root) of wheat under application of PolyPs and OrthoP at three P doses. [file 12870_2022_3683_MOESM1_ESM.docx]

**Additional File 1:**


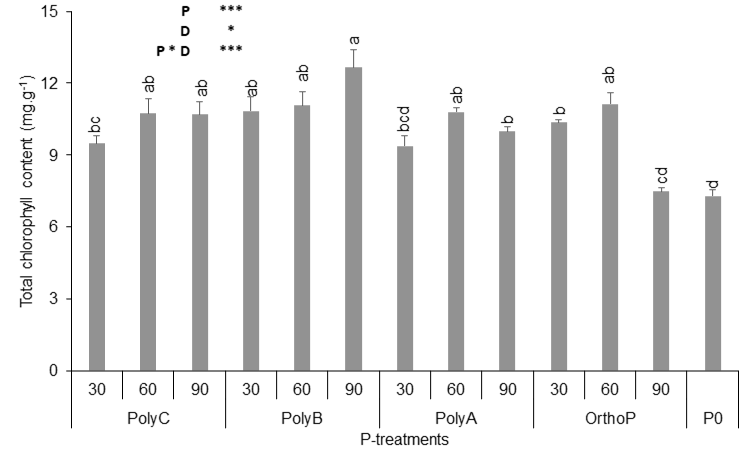


**Figure S1** Variation in chlorophyll content index of wheat under application of PolyPs and OrthoP at three P doses. Data are mean values ± SD (n=8), Different lowercase letters above the bars indicate significant differences (*p* ≤ 0.05) according to Tukey’s test. Asterisks indicate significant differences between P-fertilizers (P), P doses (D) and their interactions (P*D) (ns. not significant; **p* < 0.05; ***p* < 0.01; ****p* < 0.001).


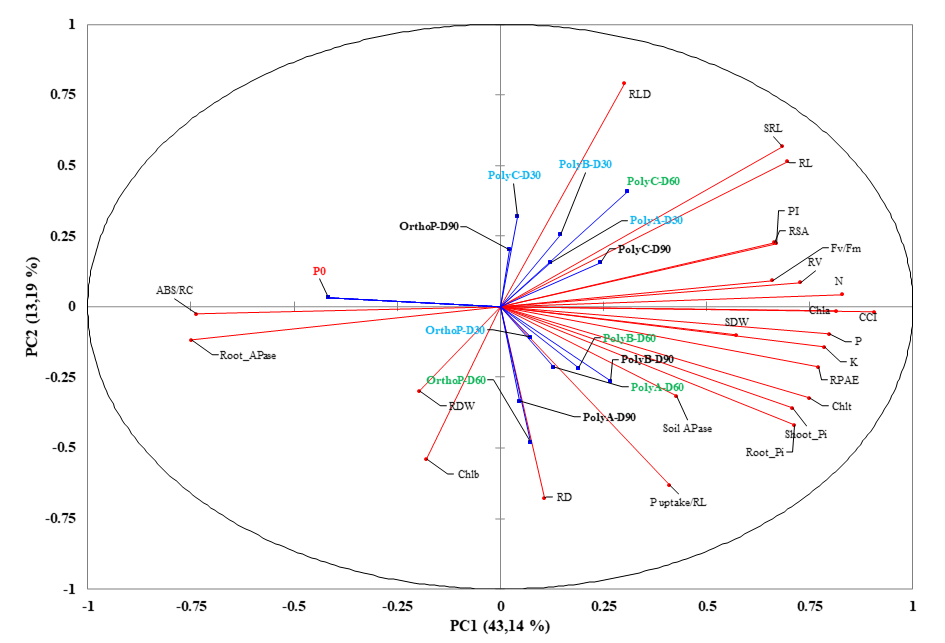


**Figure S2** Principal component analysis elaborated based on biomasses, soil acid phosphatases activity, nutrients uptake, morpho-physiological root traits and photosynthesis linked-parameters measured in durum wheat fertilized with PolyPs and OrthoP at three doses. PolyP: Polyphosphate; OrthoP: orthophosphate; RDW: root dry weight; SDW: shoot dry weight; RL: root length; RSA: root surface area; RV: root volume, RD; root diameter; SRL: specific root length; RPAE: Root P acquisition efficiency; RLD: root length density; APase: acid phosphatase; shoot_Pi: shoot Pi content; Root_Pi: Root Pi content; N, P and K: N, P and K content in the shoot; Chla : chlorophyll a content; Chlb : chlorophyll b content; Chlt : total chlorophyll content; CCI: chlorophyll content index; Fv/Fm ratio: quantum efficiency of PSII; ABS/RC: the absorption flux per reaction center and PI: performance index.

**Table S1**: Correlations (Pearson’s correlation) between root morpho-physiological traits and Pi content (shoot and root) of wheat under application of PolyPs and OrthoP at three P doses.

|  | **PolyC** | **PolyB** | **PolyA** | **OrthoP** | **P0** | **PolyC** | **PolyB** | **PolyA** | **OrthoP** | **P0** | **PolyC** | **PolyB** | **PolyA** | **OrthoP** | **P0** |  |
| --- | --- | --- | --- | --- | --- | --- | --- | --- | --- | --- | --- | --- | --- | --- | --- | --- |
|  | **Shoot Pi content** | | | | | **Root Pi content** | | | | | **Root acid phosphatase** | | | | | |
| **RL** | 0.52** | -0.67** | -0.58** | 0.43* | 0.92** | 0.65** | -0.60** | -0.35 | -0.32 | 0.85** | -0.71** | -0.06 | 0.75** | -0.74** | 0.03 |  |
| **RSA** | 0.93** | -0.46* | -0.61** | 0.31 | 0.91** | 0.40 | -0.01 | -0.41* | -0.17 | 0.82* | -0.85** | -0.33 | 0.71** | -0.79** | 0.06 |  |
| **RD** | -0.12 | 0.78** | 0.81** | 0.31 | 0.87** | -0.54** | 0.74** | 0.02 | 0.45* | 0.92** | 0.31 | -0.16 | -0.65** | 0.65** | -0.03 |  |
| **RV** | 0.79** | -0.35 | -0.42* | 0.48* | 0.92** | 0.56** | -0.40 | -0.41* | 0.01 | 0.78* | -0.81** | 0.05 | 0.58** | -0.66** | 0.07 |  |
| **RLD** | -0.17 | -0.70** | -0.61** | 0.33 | 0.67 | 0.28 | -0.56** | -0.21 | -0.55** | 0.87** | -0.08 | -0.14 | 0.77** | -0.65** | -0.23 |  |
| **SRL** | 0.58** | -0.57** | -0.49* | 0.59** | 0.89** | 0.49* | -0.64** | -0.22 | -0.28 | 0.87** | -0.69** | 0.03 | 0.63** | -0.62** | 0.16 |  |
| **Soil APase** | -0.09 | 0.69** | 0.56** | 0.11 | 0.40 | -0.40 | 0.39 | 0.00 | -0.01 | 0.19 | 0.33 | -0.36 | -0.42* | -0.14 | -0.18 |  |
| **Puptake/RL** | 0.93** | 0.94** | 0.80** | 0.12 | -0.81* | 0.19 | 0.67** | 0.35 | 0.53** | -0.89** | -0.72** | 0.02 | -0.88** | 0.65** | 0.08 |  |
| **Root_APase** | -0.88** | -0.07 | -0.89** | -0.08 | 0.08 | -0.62** | -0.32 | -0.38 | 0.25 | -0.08 | 1 | 1 | 1 | 1 | 1 |  |

Asterisks indicate significant correlation at **p* < 0.05; ***p* < 0.01; ****p* < 0.001.
